# Supplementary material for: Macromolecules with Different Charges, Lengths, and Coordination Groups for the Coprecipitation Synthesis of Magnetic Iron Oxide Nanoparticles as T1 MRI Contrast Agents
Source: Nanomaterials (Basel). 2019 May 5;9(5):699. doi: 10.3390/nano9050699 (PMC6567071; doi:10.3390/nano9050699)
Supplement: Supplementary file 1 [file nanomaterials-09-00699-s001.pdf]

Supplementary Materials: Macromolecules with Different Charges, Lengths, and Coordination Groups for Coprecipitation Synthesis of Magnetic Iron Oxide Nanoparticles as T_1 MRI Contrast Agents

Cheng Tao, Yanan Chen, Danli Wang, Yu Cai, Qiang Zheng, Lu An, Jiaomin Lin, Qiwei Tian and Shiping Yang

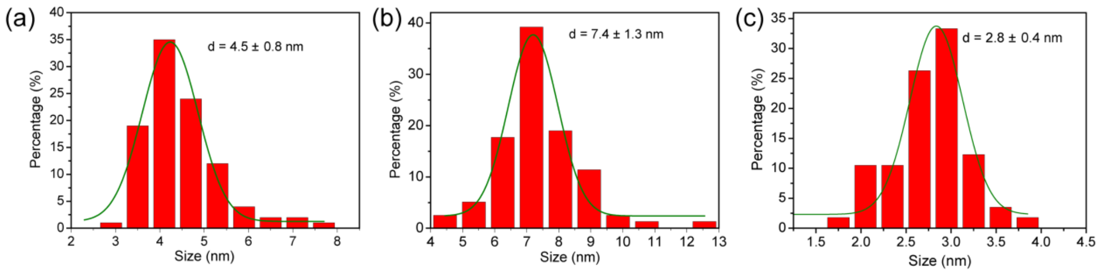

Figure S1. Particle size distribution of (**a**) Fe_3O_4-PAA, (**b**) Fe_3O_4-PAH and (**c**) Fe_3O_4-PVA acquired from the TEM images.

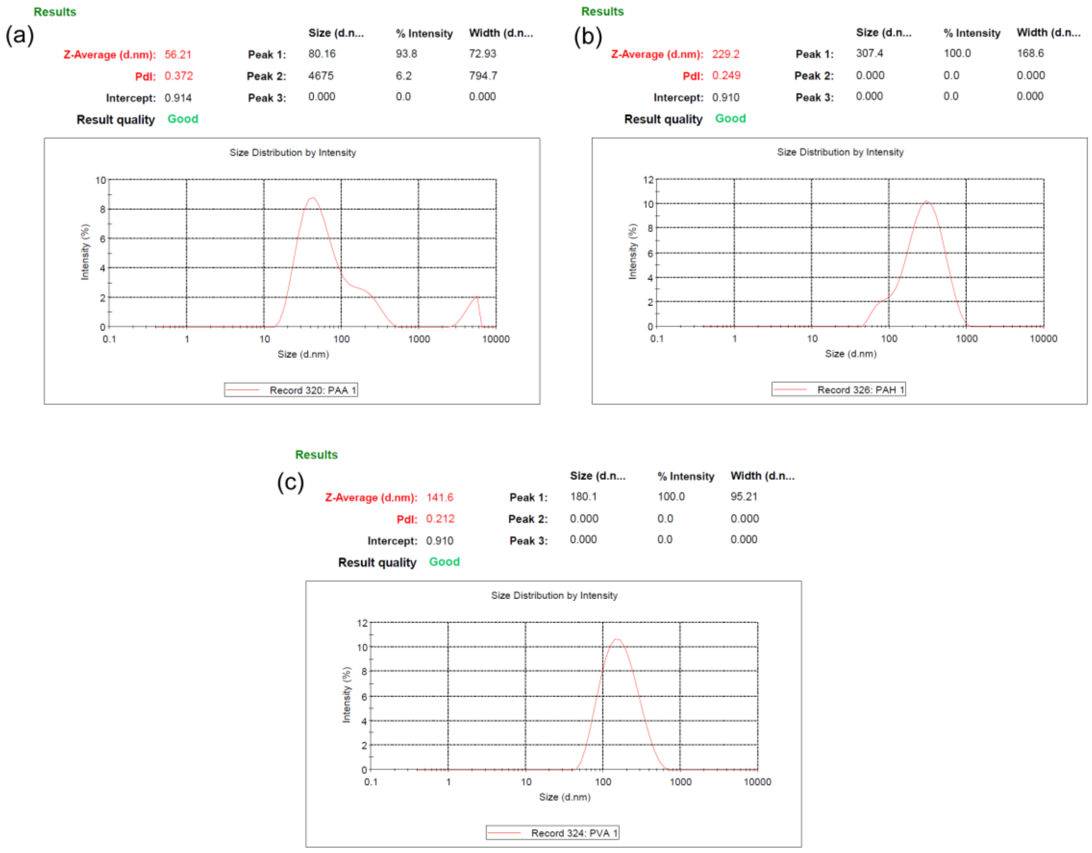

Figure S2. The hydrodynamic size profile of (**a**) Fe_3O_4-PAA, (**b**) Fe_3O_4-PAH and (**c**) Fe_3O_4-PVA nanoparticles in aqueous suspension.
